# Detection of Methicillin-Resistant *Staphylococcus aureus* among Foodborne Pathogenic Strains and Assessment of Their Adhesion Ability and Cytotoxic Effects in HCT-116 Cells

**DOI:** 10.3390/foods12050974

**Published:** 2023-02-24

**Authors:** Abderrahmen Merghni, Hiba Hamdi, Marwa Ben Abdallah, Zaki M. Al-Hasawi, Diana A. Al-Quwaie, Salwa Abid-Essefi

**Affiliations:** 1 Laboratory of Antimicrobial Resistance LR99ES09, Faculty of Medicine of Tunis, University of Tunis El Manar, Tunis 1007, Tunisia; 2 Laboratory for Research on Biologically Compatible Compounds, LR01SE17, Faculty of Dental Medicine, University of Monastir, Monastir 5000, Tunisia; 3 Laboratory of Transmissible Diseases and Biologically Active Substances, Faculty of Pharmacy, University of Monastir, Monastir 5000, Tunisia; 4 Department of Biological Sciences, Faculty of Science, King Abdulaziz University, Jeddah 21589, Saudi Arabia; 5 Biological Sciences Department, College of Science & Arts, King Abdulaziz University, Rabigh 21911, Saudi Arabia

**Keywords:** SARM, foodborne, virulence factors, HCT-116, cytotoxicity, ROS, MMP

## Abstract

*Staphylococcus aureus* is one of the high-threat pathogens equipped with a repertoire of virulence factors making it responsible for many infections in humans, including foodborne diseases. The present study aims to characterize antibiotic resistance and virulence factors in foodborne *S. aureus* isolates, and to investigate their cytotoxic effects in human intestinal cells (HCT-116). Our results revealed methicillin resistance phenotypes (MRSA) along with the detection of *mec*A gene (20%) among tested foodborne *S. aureus* strains. Furthermore, 40% of tested isolates showed a strong ability for adhesion and biofilm formation. A high rate of exoenzymes production by tested bacteria was also registered. Additionally, treatment with *S. aureus* extracts leads to a significant decrease in HCT-116 cell viability, accompanied by a reduction in the mitochondrial membrane potential (MMP), as a result of reactive oxygen species (ROS) generation. Thereby, *S. aureus* food poisoning remains daunting and needs particular concern to prevent foodborne illness.

## 1. Introduction

*Staphylococcus aureus* is one of the major human pathogens characterized by a wide range of virulence factors along with a high ability to acquire resistance to various antibiotics leading to the constant emergence of new clones [1]. For instance, methicillin-resistant *S. aureus* (MRSA) is one of the most frequent phenotypes of antibiotic resistance due to the occurrence of the clinical use of methicillin [2]. Interestingly, MRSA is no longer only considered a nosocomial pathogen associated with healthcare settings, but has also become a major cause of community-associated infections and has created several reservoirs [3]. Various surveys clearly indicate the presence of MRSA in foods that pose an immediate risk to human health. For instance, 2% to 11.9% of meat samples tested in the Netherlands were contaminated with MRSA, 5% to 7.7% in USA and Canada, respectively, and 1.2% in Tunisia [4,5,6].

*S. aureus* uses various strategic ways to maintain an infection; one of them is biofilm formation [7]. This sessile form of subsistence allows this bacterium to avoid phagocyte attacks and protects them from antibiotics and disinfectants [8]. Successful colonization of biotic surfaces such as tissue cells or abiotic ones, such as medical devices of industrial materials, is provided via cell-wall-anchored and other *S. aureus* surface proteins, many of which belong to the microbial surface components recognizing adhesive matrix molecules (MSCRAMM) family [9]. Inside the infected organism, *S. aureus* uses a wide range of toxins, enzymes and surface proteins involved in an astounding series of mechanisms to avoid host immuno-system defenses and allows the invasion of cells and propagation through tissues [10].

*S. aureus* food poisoning is induced by released enterotoxins that cause intestinal activity disruption, which is characterized by numerous symptoms such as fever, nausea, diarrhea, vomiting, etc. [11,12]. Based on antigenic heterogeneity, more than 20 enterotoxins (SEA—SElV) have been discovered [13,14]. Among them, staphylococcal enterotoxin C, causing community-associated MRSA infections and staphylococcal enterotoxin B, is associated with food poisoning [15] and involved in reducing protective T-cell responses [16].

Human cells are permanently exposed to endogenous and exogenous factors such as radiations, chemicals and pathogens (e.g., bacteria), which are leading to oxidative stress through reactive oxygen species (ROS) generation [17]. This mechanism is a result of the imbalance between the intracellular accumulation of ROS and the ability of a biological system to detoxify these reactive products [18]. Inside organisms, produced ROS such as hydrogen peroxide, hydroxyl radical and superoxide radical [19,20,21] have different negative consequences such as cycle arrest, DNA and cell membrane damage and cell alterations in apoptosis [22]. On their side, *S. aureus* can directly and indirectly (via ROS) damage host DNA of which double-strand breaks are the most deleterious [17,18,19,20,21,22,23].

Here, we characterized *S. aureus* strains isolated from food products by the screening of methicillin resistance and the assessment of adhesion ability, hemolysin and exoenzymes production as well as the cytotoxic effect in intestinal epithelial HCT-116 cells.

## 2. Materials and Methods

### 2.1. Bacterial Strains

Ten *S. aureus* strains were acquired from the Microbiology Laboratory of the Sahline Hygiene Center in Monastir, Tunisia, and have been isolated from various foodborne samples (Cake, Cheese, Milk, Sausage, Meat and Chicken). The reference strain *S. aureus* ATCC 25923 was used as a control. Bacterial identification of tested strains was confirmed with biochemical methods, by subculturing in mannitol salt agar as the selective medium and using Gram staining, catalase, coagulase and DNase tests. The strains were maintained on Luria-Bertani (LB, Liofilchem, Milan, Italy) broth, and a set preserved as glycerol stocks at −80 °C. Prior to each assay, the strains were subcultured thrice and incubated at 37 °C for 24 h to ensure optimal growth.

### 2.2. Susceptibility to Antibiotics

Antimicrobial susceptibility testing was performed with the disk-diffusion method according to the European Committee on Antimicrobial Susceptibility Testing (EUCAST). From an overnight culture of each *S. aureus* strains, a 0.5 McFarland standard bacteria solution was prepared were inoculated into a Mueller Hinton agar (MHA; Biolife, Milan, Italy) plate. The antimicrobial discs, purchased from Oxoid (Thermo Fisher Scientific, Basingstoke, UK), were penicillin G (P, 6 µg), cefoxitin (FOX, 30 µg), cefotaxim (CTX, 30 µg), kanamycin (K, 30 µg), tobramycin (TM, 10 µg), erythromycin (E, 15 µg), clindamycin (CMN, 2 µg), norfloxacine (NOR, 5 µg), ofloxacin (OFX, 5 µg), fusidic acid (FAD, 10 µg), rifampicin (RIF, 30 µg) and chloramphenicol (CHL, 30 µg). After incubation at 37 °C for 24 h, the diameters of the inhibition zones around the discs were determined.

### 2.3. Detection of Methicillin Resistance Gene

The detection of the methicillin resistance gene (*mec*A gene), was achieved by PCR amplification of specific primers 5′GTAGAAATGACTGAACGTCCGATAA-3′ (Forward) and 5′CCAATTCCACATTGTTTCGGTCTAA-3′ (Reverse). The standard thermal lyses technique was used for genomic DNA extraction from foodborne *S. aureus* isolates. Then, the PCR protocol was carried out as previously described [24]. Amplified PCR products were analyzed on a 2% (*w*/*v*) agarose gel stained with ethidium bromide (0.5 g mL^−1^) and photographed using a Gel Doc XR instrument (Bio-rad, New Castle, DE, USA). Reference strain *S. aureus* ATCC 43300 was used as a positive control (MRSA).

### 2.4. Adhesive Abilities of Tested Strains

Exopolysaccharides (Slime) production by tested isolates (*n* = 10) was assessed on Congo red agar (CRA) medium as previously described [25]. Black colonies with a rough surface on CRA plate revealed slime-producing ability of tested strains, whereas non-producing strains gave red colonies with a smooth surface.

The capacity of tested strains to form biofilms was assessed using the crystal violet assay on polystyrene-96 well plates, as previously described [26]. Reference strain *S. aureus* ATCC 25923 was used as a positive control. Biofilm formation was classified.as highly positive (OD570 ≥1), low-grade positive (0.1 ≤ OD570 < 1), or negative (OD570 < 0.1).

### 2.5. Hydrophobicity of Bacterial Cell Surfaces

The microbial adhesion to solvent (MATS) test was used to assess the cell surface hydrophobicity of *S. aureus* strains [27]. It entailed determining the cells’ affinity for apolar solvents (hexadecane). Firstly, bacterial cells were harvested by centrifugation at 7000× *g* for 5 min and resuspended in 0.01 M potassium phosphate buffer with Abs600 nm = 0.4 (10^8^ CFU mL^−1^ cell density) (Abs1) (pH 6.5). Then, bacterial suspension was vortexed for 90 s with hexadecane in a 1:6 (0.4/2.4 *v*/*v*) ratio to generate an emulsion and was left for 20 min to allow two separate phases. The aqueous phase absorbance (Abs2) was measured and the percentage of adhesion was calculated as: % adhesion = (1-Abs2/Abs1) × 100. According to Abasolo-Pacheco et al. [28], a percentage greater than 70% indicates that the tested microorganism is hydrophobic, values from 30 to 70% indicate it is weakly hydrophobic, and values less than 30% indicate it is hydrophilic.

### 2.6. Secretion of Exo-Enzymes Hemolysins

The capacity of foodborne *S. aureus* strains to generate hydrolytic enzymes was assessed after inoculating bacterial cultures on TSA medium (Biorad) supplemented with: 1% (*w*/*v*) skim milk for caséinase, 1% (*w*/*v*) gelatin for gelatinase, 5% (*w*/*v*) starch for amylase, Tween 80 for lipase and 5% (*v*/*v*) egg yolk for lecithinase [29]. The appearance of a distinct halo surrounding the colonies after 24 h of incubation at 37 °C confirms the presence of the target exo-enzyme. Regarding the hemolytic activity, it was assessed on bacteriological agar with 5% sheep’s blood [30].

### 2.7. Cytotoxicity on HCT116 Cells

*S. aureus* isolates were inoculated in tryptic soy broth (TSB, BioRad, Marnes-la-Coquette, France) and incubated in sterile 15 mL test tubes at 37 °C for 18–24 h. After centrifuging the bacterial cultures at 3000 rpm for 15 min, the supernatant was filtered through a 0.22 µm pore size filter membrane (Millipore, Merck KGaA, Darmstadt, Germany).

Human colon cancer cells (HCT116) were grown in a cell culture medium (RPMI; Sigma, Burlington, MA, USA) enriched with 10% fetal calf serum (FCS), 1% L-glutamine (200 mM), 1% penicillin (100 IU/mL) and streptomycin (100 IU/mL) at 37 °C with 5% CO_2_. On alternating days, the medium was changed. Then, on 96-well tissue culture plates, confluent monolayers of HCT116 cells were washed with PBS and 50 µL aliquots of RPMI were added to each well. Then, 50 µL of bacterial filtrates from each strain were introduced to HCT116 cell monolayers that had been previously washed in PBS and cultured for 24 h at 37 °C in 5% CO_2_. As a control, wells containing solely RPMI were used.

The MTT test was used to evaluate cell viability, as previously reported [31]. After washing with PBS, cells were treated for 1 h at 37 °C with 5-(4,5-Dimethylthiazol-2-yl)-2,5-diphenyltetrazolium bromide (MTT; Sigma). After removing the supernatants, the cells were treated with 100 µL of DMSO to dissolve the formazan crystals generated in the live metabolically active cells. The absorbance of each well of each tested isolate was measured using a microplate reader at 540 nm (Bioteck, Elx 800). The cytotoxicity percentage was computed as follows: % Cytotoxicity = (1-A540 of infected culture/A540 of control) × 100 [32].

### 2.8. Oxidative Status Evaluation

HCT-116 cells were grown at 10^5^ cells/well on 24-well culture plates (Polylabo, Strasbourg, France) for 24 h of incubation. Following that, the cells were treated for 24 h at 37 °C with various bacterial supernatants (50 µL/well). As a positive control, H_2_O_2_ (20 M) was employed. Following incubation, the cells were treated with 20 M DCFH-DA. Intracellular generation of reactive oxygen species (ROS) was measured after 30 min of incubation at 37 °C using a fluorimeter (Biotek FL 800) with an excitation wavelength of 485 nm and an emission wavelength of 522 nm. The non-fluorescent (DCFH-DA) product is converted to the highly fluorescent 2,7-dichlorofluorescein product (DCF) (lmax = 522 nm) in many processes. The fluorescent probe is degraded by intracellular esterases after diffusing across the cell membrane, converting nonfluorescent dichlorofluorescein (DCFH) to fluorescent DCF, which is contained inside the cells and oxidized by peroxides in the presence of ROS [33]. The amount of ROS produced intracellularly is proportional to the intensity of DCF fluorescence.

### 2.9. Mitochondrial Membrane Potential (MMP) Assay

The absorption of the cationic fluorescent dye rhodamine-123 was used to calculate MMP [20]. In a typical experiment, seeded cells in 96-well culture plates were treated with various *S. aureus* supernatants for 24 h, after which the cells were thoroughly washed with PBS and 100 µL of rhodamine-123 (1 mM) in PBS was reintroduced on the plates. For 15 min, cells were reintroduced to the incubator (37 °C, 5% CO_2_). The supernatant PBS (which included unabsorbed rhodamine-123) was then removed and replaced with fresh PBS. Fluorimetric detection was used to determine rhodamine-123 uptake. The results were reported as a percentage of rhodamine fluorescence uptake relative to negative control cell fluorescence.

### 2.10. Statistical Analyses 

All experiments were performed in triplicate and results were presented as mean ± SD. Statistical differences between the control and tests were determined and compared using analysis of variance (ANOVA). Differences were considered significant at *p* < 0.05.

## 3. Results

### 3.1. Susceptibility Profiles of S. aureus Strains and PCR- Detection of mecA Gene

Results of the susceptibility to antibiotics of foodborne *S. aureus* strains are summarized in Table 1. Comparing with the standard limits (CASFM), we noted that tested strains showed strong resistant to penicillin G (100%), 20% of them are resistance to cefoxitin, cefotaxime and kanamycine, 10% to tobramycine, erythromycine and clindamycine. No resistance was registered against the rest of the drugs.

**Figure 1:**
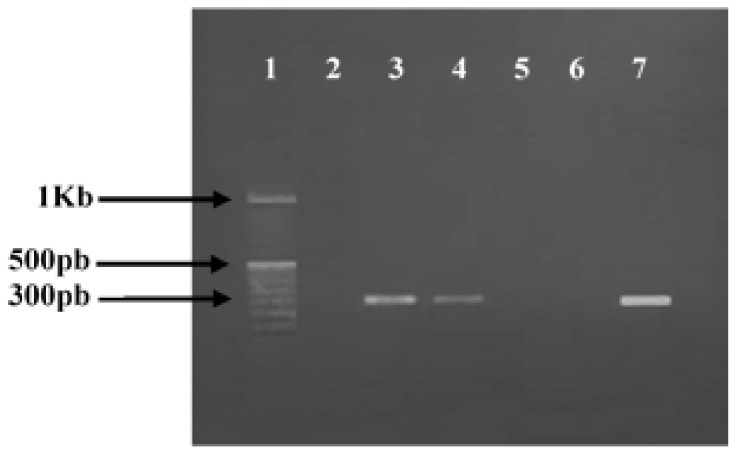
Agarose gel electrophoresis of polymerase chain reaction (PCR) amplification of *mec*A gene. Lanes 1, 100 bp DNA molecular size marker; lane 2, negative control; lanes 3–7, PCR amplicons obtained with DNA amplification of *S. aureus* strains, respectively: ATCC 43300 (Positive control); 896; 976″; 977″; 853.

Regarding the prevalence of methicillin resistance phenotype among tested *S. aureus* isolates, our results revealed that two strains (896 and 853) out of ten (20%) were found in MRSA since they are resistant to cefoxitin (FOX). Molecular confirmation of this result was carried out by PCR test revealing the detection of the *mec*A gene in the MRSA 896 and 853 strains (Figure 1; Table 1).

### 3.2. Adhesive Properties of Tested Strains

In this part of our study, we have determined the MATS of 10 *S. aureus* strains. Our data (Table 2) indicate that the affinity to hexadecane was low suggesting a hydrophilic character for all the studied strains (hydrophobicity ˂ 30%) excepting *S. aureus* 976′ that was weakly hydrophobic (30% ≤ hydrophobicity ˂ 70%).

**Figure 2:**
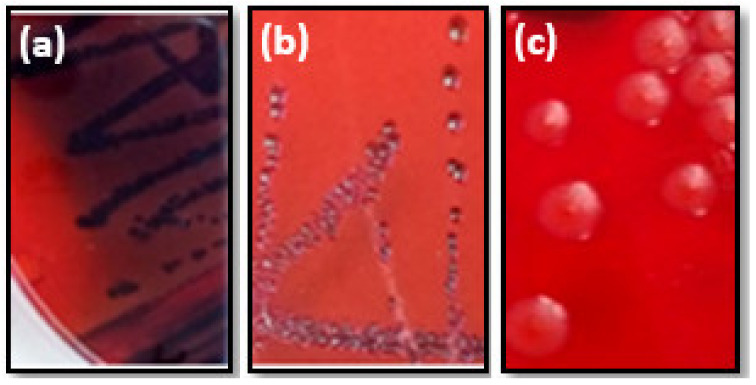
Various morphotypes of *S. aureus* strains cultivated on CRA plates. Black colonies (**a**) or red colonies with black center (**b**) indicated positive morphotype. While red colonies (**c**) bacteria revealed negative morphotype.

Regarding the ability of studied foodborne pathogens to produce exopolysaccharides (EPS) on the CRA plates, our results showed that three out of ten (30%) were slime producers developing positive and variable phenotypes (Figure 2). The other strains showed a negative phenotype (70%) (Table 2).

The result of OD570 presented in Table 2, showed that fore strains (40%) isolated from various types of foods were highly biofilm positive (OD570 ≥ 1). The rest of the strains showed a low-grade biofilm formation (0.1 ≤ OD_570_ < 1).

### 3.3. Hemolysin and Hydrolytic Enzymes Production

The resulting enzymes secretion showed that all *S. aureus* tested strains were positive for Lecithinase, Caseinase and Amylase (100%). Lipase activity was detected in eight strains (80%) and we noted that among the ten *S. aureus* strains, four were Gelatinase producers (40%). Regarding hemolysin activity, seven out of ten tested strains were beta-hemolytic (70%) (Table 3).

### 3.4. Toxicity in HCT116 Cells

**Figure 3:**
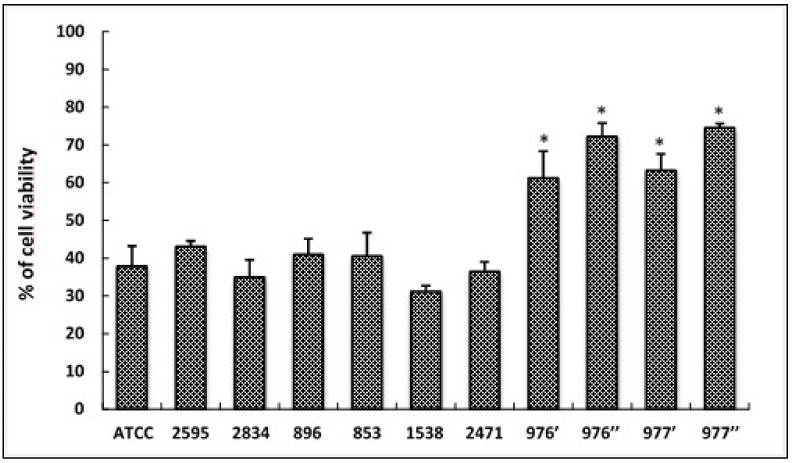
Cytotoxic effects of foodborne *S. aureus* strains on HCT116 cells treated with different bacterial extracts for 24 h. Cell viability was determined using the MTT assay and expressed as percentages of viability. ATCC referred to the reference strain *S. aureus* ATCC 25923. Data are expressed as the mean ± SD. of three independent experiments. *: Values are significantly different (*p* < 0.05) from other strains.

HCT-116 cells were treated separately with supernatant from tested *S. aureus* strains for 24 h and cell viability was determined by MTT assay (Figure 3). Our results showed that four isolates (976′; 976″; 977′; 977″) from the total tested strains induced more than 50% of cell toxicity in HCT116 cells (*p* < 0.05). The cytotoxicity potential of these strains, isolated from meat and chicken, is classified as moderate (between 50–85%).

### 3.5. ROS Generation in Infected Cells

**Figure 4:**
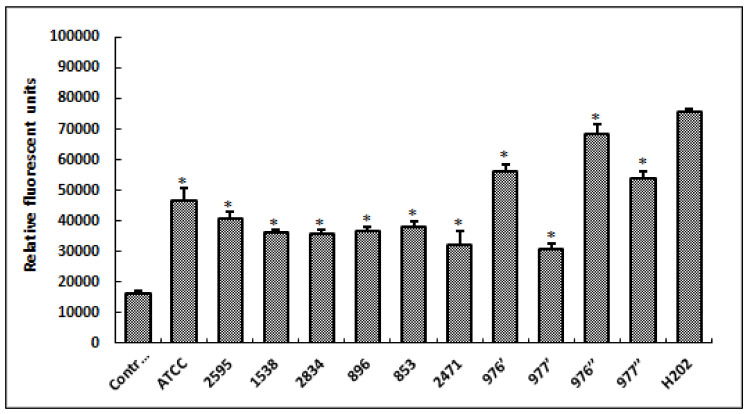
Levels of relative fluorescent DCF production, after 24 h of cell exposure to different *S. aureus* extracts. Fluorescent DCF is the result of DCFH oxidation by a variety of peroxides. H_2_O_2_, at 20 μM, was used as a positive control. ATCC referred to the reference strain *S. aureus* ATCC 25923. Data are expressed as the mean ± SD of three independent experiments. Values are significantly different (* *p* < 0.05) from control (untreated cells) and H_2_O_2_.

To check the oxidative stress status in HCT-116 cells in response to treatment with different bacterial extracts, we measured the production of fluorescent DCF (the result of DCFH oxidation by a variety of peroxides). As shown in Figure 4, our results revealed that tested extracts, from different *S. aureus* strains, induced the significant increase in ROS generation (*p* < 0.05) when compared to the control (untreated cells). Additionally, it was deduced that bacterial extracts from 976′, 976″ and 977″ strains strongly induced oxidative stress in HCT-116 cells, triggering a high production of ROS close to that of hydrogen peroxide (H_2_O_2_; positive control).

### 3.6. Loss of Mitochondrial Transmembrane Potential

**Figure 5:**
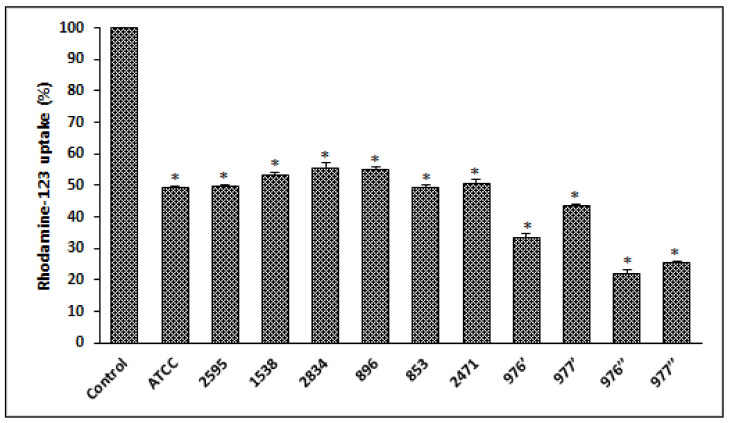
*S. aureus* extracts induces a loss of mitochondrial transmembrane potential on HCT116 cells treated for 24 h and evaluated with rhodamine-123. ATCC referred to the reference strain *S. aureus* ATCC 25923. Data are expressed as the mean ± SD of three independent experiments. Values are significantly different (* *p* < 0.05) from control (untreated cells).

After cell exposure to different bacterial extracts, from different *S. aureus* strains, a significant decrease in MMP (*p* < 0.05) was observed compared to the control (untreated cells), indicating that mitochondria were depolarized (Figure 5).

## 4. Discussion

*S. aureus* is one of the main causes of hospital and community infections [34]. The virulence of this opportunistic bacterium is a multifactorial process requiring the involvement of a variety of cellular components regulated in a perfectly coordinated manner [35]. Additionally, the unreasonable administration of antibiotics is at the origin of the incidence of MRSA infections, which have increased in recent years [36]. In the present study, we detected by simplex PCR the presence of *mec*A gene in 20% of foodborne *S. aureus* isolates, exhibiting phenotypic resistance to cefoxitin. Our finding is in agreement with previous stud dealing with the characterization of different *S. aureus* strains collected from different Tunisian food origins and showing that 1.2% of the studied strains were MRSA [6]. However, other reports showed that the percentage of MRSA prevalence can reach 38% [37]. It is well known that a wide range of staphylococcal species harbor the *mec*A gene encoding a second penicillin-binding protein (PBP2a), involved in methicillin resistance through the target modification mechanism [38]. Responsible for methicillin resistance, this gene is carried by a unique class of mobile genetic elements: the staphylococcal cassette chromosome (SCCmec) [39]. The mediators of methicillin resistance have been described with an ability to genes transfer from one species to another [40]. Particularly, the *mec*A gene has been shown to be transferred from coagulase-negative *Staphylococcus* species (SCoN) to *S. aureus* species in vivo, accounting for the emergence of more efficient MRSA clones with high adherence and invasion capabilities [41,42].

Regardless, the potential implications for MRSA reservoirs in food products demand careful monitoring of the epidemiology of this strain to design appropriate control measures before a catastrophe occurs [3]. MRSA epidemiology has increased considerably with the appearance of new strains resistant to antibiotic treatments. The majority of MRSA infections are related to biofilm formation, which is considered one of the most important virulence factors [43,44]. It is well known that most infections caused by *S. aureus* are associated with biofilm formation due to the particular characteristic of this microbial architecture allowing a high resistance to antibiotics and disinfectants, compared to planktonic forms [45]. Our results showed that 40% of isolates from various food samples were highly biofilm positive. A recent investigation carried out in Bangladesh showed that 21% of *S. aureus* isolates from different food sources are biofilm producers [46]. While other reports revealed that 72% of foodborne *S. aureus* isolates in China produced biofilms [47]. One of the major characters for bacterial adhesion and biofilm formation is cell surface hydrophobicity [48,49]. Here, we evaluated the microbial adhesion to solvent and the results revealed a hydrophilic character for the majority of studied strains, which is in agreement with another founding reporting that the *S. aureus* surface was hydrophilic [50,51].

The production of hydrolytic enzymes by *S. aureus* is an essential factor playing a critical role in virulence. In fact, they contribute to the invasion process, host tissue damage and even the propagation to other organs, regardless of its primary ecological niche [52]. Protease enzymes can change the quality of food rich in proteins resulting in a decreased shelf life of foods and their product [53]. Lipases produced from *S. aureus* could increase the rate of food deterioration through their action on lipids causing accumulation of intermediate and products that change the flavor of foods [54]. Our results showed that *S. aureus* tested strains were 100%, positive for lecithinase, caseinase and amylase and 80% for lipase. Similarly, lipase and gelatinase were identified in 82% and 88% of *S. aureus* strains isolated from Food handlers, respectively [55]. It was reported that 78%, 81% and 51% of *S. aureus* clinical isolates produced gelatinase, protease and lipase, respectively [56].

Cytotoxicity of the foodborne *S. aureus* strains was investigated using the MTT assay. After 24 HCT116 cell infections, 40% of *S. aureus* strains showed moderate cytotoxicity while the rest of the isolates (60%) showed a low cytotoxic effect on treated cells. Other findings showed that 76.2% of oral *S. aureus* strains revealed moderate cytotoxic effects on epithelial cells [57]. Numerous exoenzymes involved in the host component degradation and various toxins damaging the host tissue are secreted by *S. aureus*, along with a wide array of cell surface proteins implicated in the virulence and pathogenicity of this bacterium [58]. Through this study, a significant positive correlation (R = 0.95; *p* ˂ 0.001) was noted between bacterial exoenzymes activities and the cytotoxicity levels of foodborne *S. aureus* isolates. In fact, the qualitative assay of the soluble exo-enzymes secretion showed that the highest cytotoxic strains produced 100% of all sought enzymes.

Reactive oxygen species (ROS) are critical intermediates in oxidative metabolism in biological systems. Nonetheless, when oxidative stress occurs, ROS are produced in excess, which can harm cells by oxidizing lipids, altering DNA and destroying proteins [59]. The findings of intracellular ROS measurements clearly reveal that cell exposure to bacterial extract for 24 h significantly increases the quantity of these indicators. Under our experimental circumstances, the loss of cell viability after treatment with foodborne *S. aureus* extracts might be attributed to oxidative stress in the form of ROS production. In reality, oxidative stress is a phrase often used to describe the imbalance between ROS concentrations and cell anti-oxidative defense systems. The excessive generation of ROS changes. Excessive ROS generation affects cellular defenses, produces several harms and plays a significant role in carcinogenesis [60,61]. In terms of mitochondrial membrane potential (MMP) measurements in treated HCT116 cells, rhodamine-123 was utilized, because dynamic assessments of MMP, both in vitro and in situ, are proportional to the uptaken fluorophore [62], and MMP change is invariably related with cell death [63]. After 24 h of treatment, HCT116 cell death caused by bacterial extracts from distinct *S. aureus* strains was related to a substantial drop in MPP. It is well known that mitochondria produce the majority of ROS in cells, resulting in cell death [59]. Furthermore, it has been established that oxidative stress and MMP decline always occur concurrently [64], which is consistent with our findings.

## 5. Conclusions

In the present study, we detected methicillin resistance phenotype along with the presence of the *mec*A gene among tested foodborne *S. aureus* isolates. A high ability of biofilm formation and exoenzymes production was also registered. Additionally, tested strains exhibited cytotoxic effects on treated HCT-116 cells, predominately by ROS generation, which, in turn, induces mitochondrial dysfunction and leads to cell death. Hence, food contamination with *S. aureus* pathogen represented a real risk of acute infection, which can be avoided by hygienic quality instauration.

## Figures and Tables

**Table 1:** Susceptibility profiles of foodborne *S. aureus* strains and PCR-detection of *mec*A gene.

Code	P	FOX	CTX	K	TM	E	CMN	NORF	OFX	FAD	RIF	CHL	*mec*A
ATCC	S	S	S	S	S	S	S	S	S	S	S	S	−
2595	R	S	S	R	S	S	S	S	S	S	S	S	−
1538	R	S	S	S	S	S	S	S	S	S	S	S	−
2834	R	S	S	S	R	R	R	S	S	S	S	S	−
896	R	R	R	R	S	S	S	S	S	S	S	S	+
853	R	R	R	S	S	S	S	S	S	S	S	S	+
2471	R	S	S	S	S	S	S	S	S	S	S	S	−
976′	R	S	S	S	S	S	S	S	S	S	S	S	−
977′	R	S	S	S	S	S	S	S	S	S	S	S	−
976″	R	S	S	S	S	S	S	S	S	S	S	S	−
977″	R	S	S	S	S	S	S	S	S	S	S	S	−
% R	100	20	20	20	10	10	10	0	0	0	0	0	20

ATCC: *S. aureus* reference strain, R: Resistant, S: sensitive. P: penicillin G (6 µg), FOX: cefoxitin (30 µg), CTX: cefotaxim (30 µg), K: kanamycin (30 µg), TM, tobramycin (10 µg), E: erythromycin (15 µg), CMN: clindamycin (2 µg), NOR: norfloxacine (5 µg), OFX: ofloxacin (5 µg), FAD: fusidic acid (10 µg), RIF: rifampicin (RIF, 30 µg), chl: Chloramphenicol (30 µg).

**Table 2:** Cell surface hydrophobicity, slime production and biofilm formation ability of studied strains.

Code	Origin	Hydrophobicity		Slime Production		Biofilm Formation	
		%	State	Colonies	Phenotype	OD 570 nm	State
ATCC	-	29 ± 1.4	H. hydrophilic	Black	S(+)	1.86 ± 0.32	H.B.F
2595	Cake	4.35 ± 0.06	H. hydrophilic	Black	S(+)	0.6 ± 0.14	W.B.F
1538	Cake	18.73 ± 0.11	H. hydrophilic	Red	S(−)	1.3 ± 0.31	H.B.F
2834	Cheese	7.78 ± 0.05	H. hydrophilic	Red	S(−)	0.3 ± 0.19	W.B.F
896	Milk	19.56 ± 0.36	H. hydrophilic	Black	S(+)	2.3 ± 0.02	H.B.F
853	Milk	16.03 ± 0.15	H. hydrophilic	Red	S(−)	0.6 ± 0.18	W.B.F
2471	Sausage	28.81 ± 0.34	H. hydrophilic	Black	S(+)	2.5 ± 0.08	H.B.F
976′	Meat	38.96 ± 45	W. Hydrophobic	Red	S(−)	0.2 ± 0.07	W.B.F
977′	Meat	18.50 ± 0.12	H. hydrophilic	Red	S(−)	0.3 ± 0.15	W.B.F
976″	Chicken	23.94 ± 0.3	H. hydrophilic	Red	S(−)	0.2 ± 0.05	W.B.F
977″	Chicken	28.50 ± 0.18	H. hydrophilic	Red	S(−)	1.5 ± 0.38	H.B.F

H: Highly; W: weakly; B.F.: Biofilm forming; S(−) S(+); ATCC: *S. aureus* reference strain.

**Table 3:** Exoenzymes production and hemolysis of studied strains.

Code	Origin	Lec	Cas	Lip	Amy	Gel	Hml
ATCC	−	+	+	+	+	+	β
2595	Cake	+	+	−	+	−	α
1538	Cake	+	+	+	+	−	β
2834	Cheese	+	+	−	+	−	γ
896	Milk	+	+	+	+	−	γ
853	Milk	+	+	+	+	−	β
2471	Sausage	+	+	+	+	−	β
976′	Meat	+	+	+	+	+	β
977′	Meat	+	+	+	+	+	β
976″	Chicken	+	+	+	+	+	β
977″	Chicken	+	+	+	+	+	β
	%	100	100	80	100	40	70 β

Lec: Lecithinase; Cas: Caseinase; Lip: Lipase; Amy: Amylase; Gel: Gelatinase; Hml: Hemolysin.

## Data Availability

Not applicable.
